# Possibilities for the Biological Control of Mycotoxins in Food and Feed

**DOI:** 10.3390/toxins13030198

**Published:** 2021-03-10

**Authors:** Ksenija Nešić, Kristina Habschied, Krešimir Mastanjević

**Affiliations:** 1 Institute of Veterinary Medicine of Serbia, Food and Feed Department, Autoput 3, 11070 Beograd, Serbia; 2 Faculty of Food Technology Osijek, Josip Juraj Strossmayer University of Osijek, F. Kuhača 20, 31000 Osijek, Croatia; kmastanj@gmail.com

**Keywords:** biodetoxification of mycotoxins, detoxifying microorganisms, detoxifying enzymes

## Abstract

Seeking useful biological agents for mycotoxin detoxification has achieved success in the last twenty years thanks to the participation of many multidisciplinary teams. We have recently witnessed discoveries in the fields of bacterial genetics (inclusive of next-generation sequencing), protein encoding, and bioinformatics that have helped to shape the latest perception of how microorganisms/mycotoxins/environmental factors intertwine and interact, so the road is opened for new breakthroughs. Analysis of literature data related to the biological control of mycotoxins indicates the ability of yeast, bacteria, fungi and enzymes to degrade or adsorb mycotoxins, which increases the safety and quality of susceptible crops, animal feed and, ultimately, food of animal origin (milk, meat and eggs) by preventing the presence of residues. Microbial detoxification (transformation and adsorption) is becoming a trustworthy strategy that leaves no or less toxic compounds and contributes to food security. This review summarizes the data and highlights the importance and prospects of these methods.

## 1. Introduction

Mycotoxins are secondary metabolites synthesized by an array of fungal genera, usually *Fusarium, Penicillium* and *Aspergillus*. They are natural contaminants which commonly occur in food and feed and pose a threat to animal and human health. These hazards contaminate agricultural commodities either directly or they reach animal tissues, milk and eggs through a “carry-over” mechanism after feeding animals with contaminated feedstuffs [1,2]. From regulatory and food safety viewpoints, the most significant and prevailing types of mycotoxins are aflatoxins (AFs), zearalenone (ZEA), fumonisins (FUMs), trichothecenes (TCT) (deoxynivalenol (DON), T-2 toxin (T-2) and HT-2 toxin (HT-2)), ochratoxins (OTA), ergot alkaloids (EAs), patulin and citrinin. If these substances are present in a particularly high amount in feed and food, or in lower dosages but over a long period of time, they can cause a variety of adverse effects, from acute to chronic, both in humans and animals (Table 1).

**Table 1:** Common mycotoxins, their main producers and toxic effects.

Mycotoxin	Main Producing Fungi	Toxic Effects	Source
Aflatoxins	*Aspergillus flavus, A. parasiticus,* *A. aflatoxiformans*	Hepatotoxicity, carcinogenicity, immunosuppression	[3]
Ochratoxins	*Aspegillus ochraceus, Penicillium verrucosum*, *A. carbonarius*, *A. niger*	Nephrotoxicity, hepatotoxicity, carcinogenicity, teratogenicity, and immunosuppression	[4]
Deoxynivalenol	*Fusarium. graminearum (Giberella zeae),* *F. culmorum, F. sporotrichioides, F. tricinctum,* *F. Roseum, F. acuminatum*	Gastrointestinal toxicity, immunodepression	[5]
Zearalenone	*Fusarium. graminearum (Giberella zeae),* *F. culmorum,F. sporotrichioides*, *F. verticillioides* (*F. moniliforme*), *F. semitectum*, *F. equiseti* and *F. oxysporum*	Reproduction toxicity	[6]
Fumonisins	*Fusarium verticillioides, F. proliferatum*	Carcinogenicity, hepatotoxicity	[7]

The economic ravages induced by mycotoxins are based on increased veterinary and human health care costs, decreased livestock production, expenses of contaminated food and feed disposal, research investments and implementation of different mitigation measures to reduce the severity of mycotoxin problems, and even the possibility of fatal outcomes [8]. The World Health Organization (WHO)—International Agency for Research on Cancer (IARC) evaluated the carcinogenic potential of AFs, OTA, TCT, ZEA, and FUMs [9,10]. Escola et al. [11] emphasized that mycotoxin occurrence above the detectable levels worldwide was up to 60–80% and that even low presence should not be neglected as common mycotoxin mixtures, due to their synergism, could induce combined adverse health effects. Based on their detrimental effects on humans and animals, mycotoxin limits in several food and feed commodities have been prescribed by different national and international regulations. Nevertheless, as noted by Mastanjevic et al. [12], certain updates on legislation have to be made to provide for the health of animals and, subsequently, humans.

Mycotoxin presence in feed and food is an issue of growing concern worldwide, especially as the planet is facing the emerging effects of climate change [1]. Based on the results of the investigation of milk samples routinely used for human consumption, Mehta et al. [13] suggested a need for steps to be taken to control potential contamination of animal feed and thus to mitigate mycotoxins concentration in milk. A similar conclusion was raised by Souza et al. [14] who established good analytical methods in order to contribute to suppression of the transfer of unwanted compounds from feed to milk. The importance of laboratory control and the improvement of monitoring tests are emphasized within the work of Moradi et al. [15] considering T-2 toxin detection, or Su et al. [16] in relation to the level of DON contamination of barley and the possibility to sort the grain into different classes accordingly.

**Figure 1:**
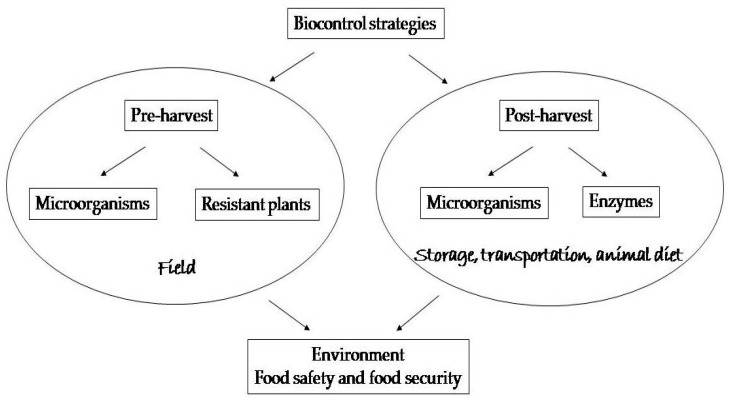
A schematic presentation of the biological strategies.

During the decades-long struggle with mycotoxin problems, many treatments have been tried: from physical, through chemical to biological. For instance, Horkey et al. [17] concluded after their investigation of effects of fungicides on mycotoxin occurrence in barley that they are partially successful and able to suppress some toxins, while not others. However, it is not just grains that are affected. The green plant mass is also at risk, as well as silage. That is why reliable solutions are being sought for these matrices as well [18,19]. Various methods have been implemented to ensure decontamination of affected commodities or to diminish the exposition to mycotoxins, but not all strategies are suitable for different purposes [20,21]. The approach based on biological agents is very promising in terms of efficiency and specificity, with the positive impact on the environment, food safety and food security. Therefore, the aim of this paper is to summarize the main achievements in this viewpoint as schematically presented in the Figure 1.

## 2. Pre-Harvest Biological Control

Pre-harvest biological control is based on in-field strategies aimed to limit contamination levels in crops intended for human and animal consumption. In general, these systems are based on prevention and meant to evade the occurrence of contamination, and to influence the predisposing factors that favor the synthesis of mycotoxins. The use of biological control agents is an up-to-date pre-harvest concept to control mycotoxin production [22].

### 2.1. Use of Microorganisms

Use of biofungicides is an approach which involves application of different microorganisms, microbial antagonists or competitors that can provide suppression of toxic fungi. This method is practiced by application of selected microbes on plants in the flowering phase to limit or completely eradicate the growth of toxigenic fungi [23]. Some microbials, as some strains of *Bacillus subtilis* can inhibit endophytic growth phase of toxin producers. Bacteria such as *Bacillus* and *Pseudomonas* and fungi belonging to the genus *Trichoderma* are the most promising biocontrol agents which act against a vast array of plant pathogens in an environmentally friendly manner [24,25].

In the literature, there are examples of fungal strains for biocontrol. Dorner and Cole [26] demonstrated that the usage of atoxigenic strains of *Aspergillus flavus* and *Aspergillus parasiticus* in soil management procedures considerably decreased aflatoxin contamination. An international collaboration between researchers gathered around the goal of solving the aflatoxin problem in Africa “gave birth” to a product called Aflasafe, which was a mixture of four atoxigenic isolates of *A. flavus*. Aflasafe strains are able to compete with toxin producers for the colonization of plant residues in soil and organic matter and now exist in multiple variants and combinations. Implementation of such products contribute to the transition from toxigenic to atoxigenic populations of *Aspergillus*, although the total amount of these fungi in the environment is not affected [27,28].

According to Cleveland et al. [29] treatment of soil with nontoxic *Fusarium verticillioides* was useful in eliminating strains that produce fumonisin and inhibited them to synthesize this toxin. Luongo et al. [30] also showed lower presence and activity of toxigenic *F. proliferatum* and *F. verticillioides* in corn residues by implementation of non-pathogenic *Fusarium* fungi. Sarrocco and Vannacci [27] described useful preharvest application of beneficial fungi in field, which afterwards resulted in a good management and prevention of accumulation of mycotoxins during storage. Later, Sarrocco et al. [31] examined the history of implementation of non-aflatoxigenic isolates of *Aspergillus flavus* aimed for prevention of aflatoxin contamination of corn and also provided an overview of the prospective usage of competitive filamentous fungi beneficial in counteracting *Fusarium* head blight in wheat and alleviating *Fusaria* toxin synthesis. Their analysis focused on the exploitation of fungi that could compete for nutrients and space (competitive exploitation) and/or fight pathogens (intervening competition). The application of such useful isolates in the field, according to their conclusion, could be a valid approach in preventing the risks associated with mycotoxin pollution of these two basic cereal plants.

The potential of fungal competitors to beat mycotoxigenic strains is, nevertheless, related to environmental conditions during their interactions. A potential limitation of the use of atoxigenic strains for the biocontrol of unwanted fungi is the risk of sexual recombination between toxigenic lines and biocontrol strains, which can lead to the emergence of hyper virulent toxigenic strains [32]. The effectiveness of mycotoxin biocontrol agents is dependent on crucial capability to infest the target substrate and to be beneficial in various surroundings, in the field or during storage, without affecting the quality of the commodity [33]. At the preharvest stage, a metagenomic approach focused on studying crop-related communities (such as those on fruits which occur naturally in field) could be helpful in detecting those beneficial isolates that could be combined and used as the agents aimed at counteracting the mycotoxin accumulation during storage [27].

### 2.2. Use of Genetically Resistant Plants

Crop damage by insects is often one of the major etiological factors in enabling toxigenic fungal infestation of plants, as these herbivores create injuries on the corn kernels and act as a vector for some varieties of fungal spores [34]. For that reason, besides agrotechnical measures, a biological strategy to plant the sorts of cereals which would be less susceptible to injuries by fungi and insects has been developed. Fungal genetics has revealed the responsible genes, pathways of mycotoxin synthesis, in particular of aflatoxins and the trichothecenes, as well as the mode of regulation of this secondary metabolism [35,36]. The development of plants resistant to the accumulation of toxins is intensively promoted in regions with wide commercialization of genetically modified crops. The success was achieved by incorporating the Bt gene into maize hybrids for the purpose of protection against insect attacks [37]. In several research trials, transgenic Bt corn has been demonstrated to decrease the accumulation of usual mycotoxins compared to non-Bt isolines. This corn contains a gene from the soil bacterium *Bacillus thuringiensis* responsible for the synthesis of a protein delta-endotoxin which is toxic to frequent *Lepidoptera* insect pests. The obtained results demonstrate the success of indirect control of vermin attacks, which are common causes of mycotoxin contamination [38].

Nevertheless, as emphasized by Munkvold [39], development of genetic resistance to *Aspergillus flavus, Gibberella zeae* and *Fusarium* spp. (particularly *F. verticillioides*) in corn is a high priority. He also stated that Bt maize is efficient in the reduction of fumonisin occurrence, but is less successful in minimizing deoxynivalenol presence. This discrepancy mirrors various pathogen and disease models as deoxynivalenol is associated with *Gibberella* ear rot, whereas fumonisin synthesis is related to *Fusarium* ear rot, and the incidence of *Gibberella* ear rot is not as strongly affected by insect damage as is fumonisin formation. The total benefit of reducing fumonisins and aflatoxins by Bt corn in the United States is estimated at USD 23 million annually [37], while the new evaluation of the decrease in aflatoxin accumulation due to planting Bt corn reaches USD 120 to USD 167 million per year in over 16 states on average [38].

Continuous attention is being devoted to the uprising of transgenic plants which show resistance against various diseases. Fungal and mycotoxin counteraction strategies, as part of the plant-disease management via genetic engineering, are being pursued intensively in three basic ways: (a) detracting infestation by the pathogen, (b) inserting detoxifying genes, or (c) minimizing mycotoxin accumulation by influencing the biosynthetic pathway [39]. This is considered to be the latest approach to reduce the dependency on harmful synthetic fungicides. The current need is to identify genes across species to encourage the search for variation against biotic stress. During the last twenty years, remarkable efforts have been made towards implementation of genetic engineering in plant-disease management. Additionally, various molecular methods have appeared to unravel multiple plant-pathogen combinations and connected prospect genes responsible for disease resistance. Such genes have been recognized and estimated in crop improvement programs by transformation [40]. Observing recent events, which have resulted in new active resistance genes, it is motivating for emerging approaches to develop new specific resistance genes by gene modification [41].

## 3. Post-Harvest Biological Control

Although infestation by toxigenic fungi and mycotoxin synthesis are inevitable under certain environmental circumstances, their prevention is the preferred goal. Therefore, appropriate pre-harvest practices and initially good quality of cereals represents the first combat line, but post-harvest control systems are essential to diminish the final contamination of various agricultural products. A number of strategies are available for the mycotoxin degradation and/or fungal inactivation. The main advantages of biological control are that it proved to be more effective, specific, irreversible and environmentally friendly [22]. The main biological methods based on the use of microbiological agents and enzymes in food and feed will be further discussed.

### 3.1. Use of Microorganisms

Biodetoxification is a relatively new strategy for mycotoxins reduction via nonpathogenic microbes or their enzymes via catabolic processes. These germs not only lead to reduction or suppression of toxins to no or less toxic compounds, but are also considered as basically safe as they provide useful end products through the mechanisms of biodegradation or bioadsorption. The antagonistic outcome of probiotics on toxigenic fungi arises from competition for the living space and nutrients that are necessary for growth, metabolism, the parasitism and parasitic function on pathogen fungi by forming a biofilm and also making a defensive response during the release of free oxygen radicals [42]. The use of different microorganisms (bacteria, yeast and fungi) for the control of common mycotoxins have been summarized by Taheur et al. [43] and presented here within the Table 2.

#### 3.1.1. Bacteria

Development of bacteria capable for biotransformation of mycotoxins into nontoxic metabolites, which exert its function within the intestinal tract prior to the resorption of the mycotoxins, began in the 1980s. The first were *Flavobacterium aurantiacum* with the capacity to detoxify aflatoxins; *Phenylobacterium immobile* proved to degrade ochratoxin A and *Gliocladium roseum* which detoxified zearalenone via ring opening with subsequent decarboxylation [44]. Detoxification of aflatoxin B1 by *Enterococcus faecium* is a consequence of the mycotoxin adherence to the bacterial cell wall components, a modus that has been further set up through various studies. Bacterial cell wall peptidoglycans and polysaccharides were demonstrated to be constituents responsible for mycotoxin adsorption by the aid of microorganisms [45].

Considering effects of trichothecenes, it is well known that the 12,13-epoxide ring is in charge of their toxic activity, so the removal of this epoxide group causes a significant loss of toxicity [46]. *Eubacterium* BBSH 797 was the first isolated individual bacterial strain which was capable of biotransforming the epoxide group of trichothecenes. This strain, which originates from bovine rumen fluid, by its epoxidase enzymatically reduced deoxynivalenol (DON) to the non-toxic metabolite de-epoxy-deoxynivalenol (DOM-1). It was the first microorganism applied as a mycotoxin deactivating additive in feed. Regarding the microorganism DSM 11798 *Genus nov. species nov.* (BBSH 797) product, EFSA (European Food Safety Authority) delivered a positive opinion on its safety for the target animals (pigs and avian species), consumer, user and the environment, when used under the proposed conditions [47,48]. The appropriate implementing regulations were established in 2016 and 2017 [49,50]. Aerobic oxidation and epimerization of DON at the C3 group performed by multiple soil microorganisms, mainly belonging to the Gram-negative *Devosia* genus, was reviewed by Hassan and Zhou [51]. A novel bacterium *Eggerthella* sp. DII-9 was isolated by Gao et al. [52] from chicken intestines, who also determined its ability to biotransform DON, HT-2, T-2 triol and T-2 tetraol.

Several researchers have demonstrated the biodetoxification of mycotoxins using probiotic lactic acid bacteria [53,54,55,56]. Probiotics can remove these contaminants by biodegradation or bioadsorption pathways. Biodegradation is irreversible and of longer duration compared to bioadsorption, but it can modify toxin structure and also result in unwanted metabolites (e.g., aflatoxicol from aflatoxin B1), which could be detrimental for the host. Bioadsorption assumes quick direct binding of toxin which might be simply released and depends on the bacterial affinity toward toxin [57]. *Bacillus* and *Brevibacterium* species have been studied for degradation of different mycotoxins: aflatoxin, zearalenone, deoxynivalenol, ochratoxin and patulin. These mycotoxins could be also adsorbed by lactic acid bacteria of *Lactobacillus*, *Bifidobacterium* and *Lactococcus* strains, but in a different adsorption range [53,58].

#### 3.1.2. Yeast

As a way of biological control, probiotic yeasts or products that contain yeast cell wall have also been implemented to defeat mycotoxins. A variety of yeast strains proved to be effective in transformation of toxins to non-toxic or at least less-toxic products, while some of them suppress the development of filamentous fungi. The utilization of yeasts in different technological procedures may have a direct inhibitory effect on the synthesis of toxins by certain fungi, whereas several species possess the ability to accumulate mycotoxins from agricultural products, thereby successfully detoxifying them [59].

The advantage of these microorganisms is that they have mere nutritional needs and are able to settle on dry surfaces over longer periods of time, as well as that they tolerate various pesticides used in the post-harvest conditions [60]. Contrary to many mycelial fungi, yeasts mostly do not produce allergenic spores or mycotoxins and they are also not capable of synthesizing antibiotic metabolites, which can be produced by bacterial antagonists [61,62]. Additionally, they can rise fast on affordable substrates in fermenters and are therefore convenient for production in large amounts [63]. Utilization of yeasts is harmless to humans, animals, host plants or the environment, and it is unlikely that the target organisms will generate resistance [64].

Four strains of yeasts: *Saccharomyces cerevisiae* AUMC 3875, *Pichia anomala* AUMC 2674, *Pichia guilliermondii* AUMC 2663 and *Candida krusei* AUMC 8161 were chosen by Zohri and Abdel-Kareem [65] as agents for biocontrol of growth and production of mycotoxins by 11 different toxigenic fungal isolates. In their experiment, *Candida krusei* AUMC 8161 absolutely prevented the development and production of toxins of all 11 investigated toxigenic isolates. *Pichia anomala* AUMC 2674 fully suppressed the development and synthesis of toxins originating from six mold isolates and extremely decreased the growth as well as the production of toxins from other experimental toxigenic fungi. *Pichia guilliermondii* AUMC 2663 greatly diminished the production and growth of toxins synthesized by 11 toxigenic fungi. *Saccharomyces cerevisiae* AUMC 3875 absolutely prevented development of five fungal isolates and greatly decreased the growth of other molds.

*Saccharomyces cerevisiae* is considered to be a probiotic yeast which can, according to Liu et al. [66], remarkably decompose deoxynivalenol (DON) and decrease the extent of lactate dehydrogenase (LDH) release in cells stimulated by DON. Success in alleviating the effects of ochratoxin A and aflatoxin B1 by utilization of yeast *Saccharomyces cerevisiae* cell wall in chicken diets has been recently reported by Mendieta et al. [67]. Efficacy of this yeast to remove patulin in fermented foods by physical adsorption has also been proven [68]. *Kluyveromyces marxianus* were used to bind aflatoxin B1, ochratoxin A and zearalenone, while authors demonstrated that mycotoxins can be bound especially by the *Candida utilis* cell [69]. In a different trial, the yeast *Yarrowia lipolytica* reduced the quantity of ochratoxin A to approximately half of the starting concentration applied in the culture [70]. More than 50% degradation of patulin by *Rhodotorula mucilaginosa* (*R. mucilaginosa* JM19) indicates the usefulness of this yeast in foods and raw materials [71].

#### 3.1.3. Fungi

Concerning the fungi and their detoxifying abilities, it was demonstrated that those species capable of synthesizing mycotoxins could often also degrade them. Therefore, the application of nontoxigenic strains of *A. parasiticus* and *A. flavus* on plants (maize, peanuts, pistachio and cotton) has achieved exceptional results in the elimination of aflatoxins. This is due to the fact that these fungi commonly have the ability of degradation and probably conversion and utilization of degradation products [72]. Usage of high dosages of non-toxigenic inoculants in the soil around developing crops provides competition with toxigenic strains for infestation sites on the growing plant. This methodology also brings positive effects during storage as competitive elimination in the field transforms into a reduced risk of toxin presence in the storehouses and transportation. In this way, less toxin-producers move into the storage and the applied biocontrol agents persist on the crop until its final use [73].

There are also other fungi, like *Rhizopus*, *Trichoderma*, *Clonostachys* and *Penicillium* spp., that might fit for mycotoxin biocontrol [73]. It was demonstrated by Hackbart et al. [74] that *Rhizopus oryzae* and *Trichoderma reesei* reduce aflatoxins AFB1, AFB2, AFG1, AFG2 and AFM1. *Trichoderma* strains have also exerted considerable antibiosis and parasitism ability, making them suitable to be used as mycoparasites against toxigenic *Fusarium* isolates for preventing their growth by forming coils around the *Fusarium* hyphae and penetrating it [75]. As with other biocontrol methods, the concept is to have such formulation able to oppose the mycotoxin-producing strains and make a toxin-free product. Non-toxigenic *Fusarium verticillioides* appeared to be a promising species against fumonisin-forming *Fusarium* strains, but at the same time regrettably it is a plant pathogen [76].

In vitro experiments with inoculation of *Microsphaerosis* species on maize and wheat grains reduced production of *Gibberella zeae* ascospore by 73%, while also in another trial, under glass house conditions, with *Phoma betae* inoculation on wheat ears the prevalence of *Fusarium* head blight decreased up to 60% [77]. As summarized by Vankatesh and Keller [78], there are other fungi with mycotoxin transforming properties based on different mechanisms. Fungi *Clonostachys rosea* has been shown to synthesize lactonase, a zearalenone-specific enzyme which catalyzes the hydrolysis of the lactone ring followed by spontaneous decarboxylation [79]. Conversion of zearalenone into ZOM-1, characterized by the opening of the ring structure at the ketone group positioned at C6′, reported to be provided by *Trichosporon mycotoxinivorans* [80].

There are some doubts and even counter arguments considering the usage of some fungi for the wide control of mycotoxins. For instance, non-aflatoxigenic *Aspergillus* AF36 was officially applied for biological control treatments to alleviate aflatoxin problems in the USA, but it also produced cyclopiazonic acid (α-CPA), which is a proven inhibitor of ATP-ase enzyme and possessed the ability to impair physiological muscle function (contractions and relaxations). Consequently, instead of AF36 other non-aflatoxigenic strains, unable to synthesize α-CPA, are currently in use as agents for biocontrol [81]. Available data show that *A. flavus* strains can produce a multitude of different metabolites with unrevealed toxicological outcome, such as aflavinine, aspertoxin, aflatrem, kojic acid, leporin C, paspalinine and sterigmaticystin [82]. There is also an evidence that a nontoxigenic strain can transform into a toxigenic one through sexual reproduction. Therefore, it is necessary to have complete insight into the action of the agents and all safety issues must be considered before their usage.

### 3.2. Use of Enzymes

Significant efforts have been recently invested to find enzymes able to degrade and metabolize mycotoxins and thus provide adequate biotransformation solution to the mycotoxicology problems. Such biotechnological methods, which are highly specific, generate harmless products, and preferably lead to total detoxification while acting environmentally friendly, are a primary goal. The main conversion paths are hydroxylation, hydrogenation, hydrolysis, oxidation, esterification, glucuronidation and glycosylation, de-epoxidation, methylation, sulfation, demethylation and deamination [83], which depends on the type and nature of the mycotoxin. Many promising solutions have been reported targeting aflatoxins, fumonisins and ochratoxins [84,85,86,87,88,89,90,91]. Deoxynivalenol (DON) due to its widespread (globally the most commonly detected agricultural mycotoxin) and its chemical nature (small polar moiety), appeared to be the most difficult target to develop agents able to irreversibly bind it. This made it a challenging task for numerous innovative investigations designed to discover feasible and sustainable biological degrading solutions [92]. The mechanism of the enzyme action towards zearalenone (ZEN) has been studied in detail by several scientific teams [93,94] and developed detoxification strategies are intended to disrupt its estrogenic activity. The prevalent ZEN detoxifying mode described so far is cleavage of lactone ring, which is catalyzed by esterases. The reaction is irreversible since the resulting hydroxyketones spontaneously decarboxylate [95]. Ferrara et al. [96] have shown that a function-driven methodology which involves metagenomic analysis represents a potent researching tool aimed to reveal novel enzymes powerful in degradation of mycotoxins. They discovered the role of two new carboxylesterase genes belonging to *Dysgonamonadaceae bacterium* and *Peptococcaceae bacterium* assumed to be involved in fumonisin degradation. Enzymes for the control of common mycotoxins, accompanied with their producers, as summarized by Loi et al. [95] are given within the Table 3.

As usually several mycotoxins simultaneously contaminate commodities, Lyagin and Efremenko [97] suggested development of biocontrol agents which contain several efficient enzymes. To select proper enzymes for such combinations precisely, both a thorough understanding of catalytic processes and proper analysis of enzyme properties are required. Useful solutions for this purpose would be enzymes that can degrade several mycotoxins at the same time, but only a limited number can answer this task. There are cytochromes (able to modify aflatoxins, trichothecenes and sterigmatocystin), aflatoxin oxidase AFO (working on aflatoxins and sterigmatocystin) and aldo-keto reductase AKR18A1 (performing reduction of trichothecenes and zearalenone) among them. In addition, there are numerous mycotoxins (like sterigmatocystin and ergot alkaloids) which can be detoxified by a very limited number of enzymes, if there is such possibility at all, while also more than a half of enzymes (lyases, isomerases, ligases and translocases) are unable or unknown to be able to modify mycotoxins [97].

Both from technological and economic points of view, a use of enzymes is beneficial. Reduced effectiveness due to matrix influence could be present. The physicochemical properties of food (fat content, moisture, acidity, texture) significantly affect the outcome of the detoxifying process. Furthermore, inhibitory components could be present in raw materials and the possibility to have masked forms of mycotoxins may restrict their bioavailability for the enzymatic catalysis. Such circumstances might require pretreatments, additional finance and time, which must be carefully considered when establishing industrial utilization [95]. The use of biological agents as feed additives could also be quite limited. To make it widely applicable more understanding is needed about the conversion procedures, the toxicological characteristics of the products obtained by transformation, the influence of the conversion on nutritional value of feed and on animal safety. Such feed additive must be harmless and stable in the digestive tract of animals [98]. An appropriate technological prescription of enzyme application is necessary in order to preserve its efficiency.

It is also important to have proper legislation, both for food and feed, which foresees the possibility of detoxifying treatments. The European Community set the regulation on food enzymes in 2008 [99]. This regulation covers “enzymes added to food to perform a technological function in the manufacture, processing, preparation, treatment, packaging, transport or storage of such food, including enzymes used as processing aids”. All enzymes included in this regulation are considered as processing aids, excepting invertase and lysozyme, which belong to the group of additives. Eligibility criteria for detoxifying treatments, including biotransformation, have been established for materials intended for animal nutrition in 2015 [100]. EU Regulations have been established regarding enzyme fumonisin esterase as feed additive for pigs and poultry [101,102,103] and EFSA gave its scientific opinion in 2020 [104] on safety and efficacy of fumonisin esterase from *Komagataella phaffii* DSM 32159 as a feed additive for all animal species, in accordance with Regulation (EC) No 1831/20031 which establishes the rules governing the community authorization of additives for use in animal nutrition [105].

## 4. Conclusions

Biological control agents should be affordable to food and feed producers and composed in a way that makes products easy and safe to handle. The efficacy might be enhanced by the selection of more efficient strains of microorganisms, gene manipulations, combination of more ingredients and inclusion of other synergistically acting bio-products. The biocontrol of mycotoxins is an approach with a bright future, although it will not be self-sufficient. It should be implemented in connection with good agricultural practices and coupled with good postharvest management, especially sorting and suitable storage. Most questions concerning safety, sustainability and the impact on the ecosystem of biological strategies are asked from stakeholders in industry, academia and local governments, and uppermost from the consumers. Therefore, many tests should be conducted and the results evaluated with the aim to eliminate any suspicions on possible adverse effects on plant, animal and human health and the environment. Without a doubt, before making the final choice of method, all issues must be resolved and a complete risk assessment carried out.

## Figures and Tables

**Table 2:** The use of microorganisms (bacteria, yeast and fungi) for the control of common mycotoxins (Adapted from Taheur et al. [43]).

Mycotoxins	Microorganisms
Aflatoxins	*Lactobacillus plantarum* LOCK 0945, *L. brevis* LOCK 0944, *L. paracasei* LOCK 0920, *L. kefiri*, *Bacillus pumilus*, *Bacillus subtilis* ANSB060, *Kazachstania servazzii, Acetobacter syzygii, Rhodococcus erythropolis, Pseudomonas putida, Mycobacterium fluoranthenivorans* sp. *nov*. DSM 44556T, *Streptomyces lividans* TK 24, *Saccharomyces cerevisiae, Pichia anomala, Fusarium aurantiacum* strain NRRL-B-184, *Pseudomonas putida, Mycobacterium fluoranthenivorans* sp. *nov.* DSM 44556T, *Streptomyces lividans* TK 24, *Flavobacterium aurantiacum*
Ochratoxin A	*L. acidophilus* VM 20, *L. bulgaricus, L. helveticus, L. rhamnosus* GG, *B. lichniformis, B. subtilis, Bifidobacterium animalis* VM 12, *Brevibacterium, Cupriavidus basilensis* ŐR16, *Pediococcus parvulus, B. amyloliquefaciens* ASAG1, *S. cerevisiae* O11, *S. bayanus, Yarrowia lipolytica*
Zearalenone	*B. licheniformis* CK1, *B. pumilus* ES-21, *B. subtilis, L. mucosae* lm4208, *L. rhamnosus, P. otitidis* TH-N1, *Rhodococcus, Lysinibacillus* sp., *Geobacillus* and *Tepidimicrobium*
Trichothecenes (DON, T-2/HT-2)	*Nocardioides* and *Devosia, Lactobacillus sakei* KTU05-6, *Pediococcus acidilactici* KTU05-7, *Pediococcus pentosaceus* KTU05-8, KTU05-09 and KTU05-10, *Eggerthella* sp. DII-9

**Table 3:** Enzymes for the control of common mycotoxins (Adapted from Loi et al. [95]).

Mycotoxin	Enzyme	Producer
Aflatoxin	Aflatoxin oxidase enzyme (AFO) (EC 1.1)	*Armillariella tabescens*
	Peroxidase (EC 1.11.1.7)	Horseradish (*Armoracia rusticana*)
	Laccase (EC 1.10.3.2)	*Trametes versicolor* (Sigma-Aldrich, St. Louis, MO, USA)
	Laccase (EC 1.10.3.2)	*Streptomyces coelicor*
	F420H2-dependent reductases (E.C. 1.5.8)	*Mycobacterium smegmatis*
	Mn peroxidase (EC 1.11.1.7)	*Pleurotus ostreatus*
	Aflatoxin degradation enzyme	*Pleurotus ostreatus*
	Myxobacteria aflatoxin degrading enzyme (MADE)	*Myxococcus fulvus* ANSM068
	Laccase (lac2) (EC 1.10.3.2)	*Pleurotus pulmonarius* (ITEM 17144)
	Ery4	*Pleurotus eryngii* (PS419)
Fumonisin	Carboxylesterase and aminotransferase (E.C. 3.1.1, E.C. 2.6.1)	*Sphingomonas* sp. ATCC55552
	Carboxylesterase B and aminotransferase (E.C. 3.1.1, E.C. 2.6.1/FJ426269.1)	*Sphingopyxis* sp. MTA144
	Fumonisin esterase (E.C. 3.1.1.87)	*Sphingopyxis* sp. MTA144
Trichothecenes	Cytochrome P450 system (Ddna + Kdx + KdR) (E.C. 1.14 AB744215.1 AB744217.1) (DON; NIV)	*Sphingomonas* sp. strain KSM1
	UDP-glycosyltransferase (AC006282)	*Arabidopsis thaliana*
Zearalenone	Laccase (EC 1.10.3.2)	*Trametes versicolor* (Sigma-Aldrich, USA)
	laccase (EC 1.10.3.2)	*Streptomyces coelicolor*
	Lactono hydrolase (E.C. 3.1.1)	*Clonostachys rosea*
	2cys-peroxiredoxin (EC 1.11.1.15)	*Acinetobacter* sp. SM04
Ochratoxin	Carboxypeptidase A: CPA (EC 3.4.24)	Bovine pancreas
	Carboxypeptidase Y: CPY (EC 3.4.16)	*Saccharomyces cerevisiae*
	Lipase (EC 3.1) Protease A (EC 3.4) Amidase 2 (EC 3.5)	*Aspergillus niger*

## Data Availability

Not applicable.
